# A Comparative Study of Human Saposins

**DOI:** 10.3390/molecules23020422

**Published:** 2018-02-14

**Authors:** María Garrido-Arandia, Bruno Cuevas-Zuviría, Araceli Díaz-Perales, Luis F. Pacios

**Affiliations:** 1 Centro de Biotecnología y Genómica de Plantas (CBGP, UPM-INIA), Campus de Montegancedo-UPM, 28223 Madrid, Spain; maria.garrido@upm.es (M.G.-A.); bruno.czuviria@upm.es (B.C.-Z.); araceli.diaz@upm.es (A.D.-P.); 2 Departamento de Biotecnología-Biología Vegetal, ETSIAAB, Universidad Politécnica de Madrid (UPM), Ciudad Universitaria, 28040 Madrid, Spain

**Keywords:** saposins, lipid-antigens, protein-ligand interactions, protein-membrane interactions, electrostatic potentials, molecular dynamics

## Abstract

Saposins are small proteins implicated in trafficking and loading of lipids onto Cluster of Differentiation 1 (CD1) receptor proteins that in turn present lipid antigens to T cells and a variety of T-cell receptors, thus playing a crucial role in innate and adaptive immune responses in humans. Despite their low sequence identity, the four types of human saposins share a similar folding pattern consisting of four helices linked by three conserved disulfide bridges. However, their lipid-binding abilities as well as their activities in extracting, transporting and loading onto CD1 molecules a variety of sphingo- and phospholipids in biological membranes display two striking characteristics: a strong pH-dependence and a structural change between a compact, closed conformation and an open conformation. In this work, we present a comparative computational study of structural, electrostatic, and dynamic features of human saposins based upon their available experimental structures. By means of structural alignments, surface analyses, calculation of pH-dependent protonation states, Poisson-Boltzmann electrostatic potentials, and molecular dynamics simulations at three pH values representative of biological media where saposins fulfill their function, our results shed light into their intrinsic features. The similarities and differences in this class of proteins depend on tiny variations of local structural details that allow saposins to be key players in triggering responses in the human immune system.

## 1. Introduction

Antigen presentation molecules are key players in innate and adaptive immune responses. Whereas the major histocompatibility complex class I and II proteins present peptide antigens to T cells, Cluster of Differentiation 1 (CD1) molecules can bind a great diversity of lipidic ligands and are thus responsible for presenting lipid antigens to T cells and a variety of T-cell receptors [1,2,3]. Exogenous lipids are transported to different endocytic compartments according to their length after incorporation into the membrane. CD1 molecules traffic through those compartments sampling antigens in the endocytic pathway. However, the nonpolar nature of lipids implies that their extraction from membranes and mobilization across the aqueous endocytic environment will have unfavorable energetics, which poses the necessity to require the assistance of molecular mediators. Saposins are the best candidates for this role [4]. Saposins (for “sphingolipid activator proteins”, or SAPs) are small (8–11 kDa), acidic, non-enzymatic, heat-stable, protease-resistant, lipid-binding proteins known to assist in CD1 lipid loading in endosomal compartments [4,5]. Four saposins named A, B, C, and D are generated through proteolytic cleavage from a single precursor protein, prosaposin, that is post-translationally processed to the four mature saposins [6]. Prosaposin deficiencies in mice result in defects in CD1-mediated antigen presentation to natural killer T- (NKT) cells [5] and studies on human patients with prosaposin mutations indicate that this protein may also exert neuroprotective actions [7].

While it is well established that saposins facilitate the transfer of lipid antigens from membranes into CD1 antigen-presenting proteins, the complete mechanisms are not yet understood. All saposins assist in this process but they appear to have different abilities to load particular lipids into different CD1 isoforms and each of the four saposins has a distinct role in promoting hydrolysis of sphingolipids [8]. Although relevant details on their processing of lipids are still lacking, direct interactions between saposins and lipids are known to occur. In this regard, two fundamentally distinct lipid loading mechanisms have been proposed depending on whether short or long chain lipid antigens are loaded. In one mechanism (shown by saposin C), the saposin embeds into the membrane disrupting the lipid lattice of membrane bilayers and orients CD1 via protein-protein interactions for lipid loading at the membrane surface. In a second mechanism (shown by saposins A and B), the saposin extracts lipids from the membrane through a tweezer-like process by inserting hydrophobic residues exposed at surface loops around polar headgroups of phospho- and glycolipids, and then form saposin-lipid soluble intermediates that load CD1 molecules, away from the membrane surface [8,9].

Genetic anomalies in saposins lead to pathological accumulation of specific sphingolipids. Saposin A deficiency causes galactosylceramide lipidosis, saposin B defects lead to leukodystrophy resulting in sulfatide accumulation, saposin C anomalies produce glucosyl ceramide lipidosis, and saposin D genetic inactivation causes ceramide accumulation [10]. In vivo and in vitro assays show that all saposins facilitate CD1 lipid loading although with different abilities to load particular lipid antigens into different CD1 isoforms [4,5,8]. Saposin A in the presence of a zwitterionic detergent (lauryldimehtylamine-N-oxide) forms lipoprotein arrangements in which two chains of saposin A in an open conformation trap 40 detergent molecules in the form of bilayer-like discs although these discs show limited solubility for sphingolipids, phospholipids, and cholesterol [11]. Saposin B extracts lipid antigens from membranes and in the form of soluble complexes transports the lipids to recipient CD1 [12]. These complexes are protein dimers in which the monomers adopt an open, V-shaped conformation that create a large hydrophobic compartment for lipid binding [13]. Saposin C enables glucosylceramidase access upon modifying membranes containing glucosylceramide by a detergent-like action at the membrane surface and promotes association of the enzyme with membranes containing phospholipid [9,14,15,16]. Saposin D is required to activate acid ceramidase for breakdown of ceramide into fatty acid and sphingosine in lysosomes [17].

In addition, saposins have been reported to unload lipids bound to CD1d [5], a result that led to the proposal of a “tug-of-war” model for antigen loading and exchange. According to this model, the saposin would load or extract lipids from CD based on the affinity of both proteins for the same lipid [5]. Although no structural data supporting this hypothesis are available yet, it is accepted that a physical interaction between saposins and CD1 molecules does exist. It was found by co-immunoprecipitation that saposins C and D interact directly with CD1b and that saposin A and CD1d with the lipid harbored in its binding site form a complex [5,18].

The processing and loading of lipid antigens onto CD1 molecules require localization to endosomal and lysosomal subcellular compartments in which the environment is acidic. In fact, the pH decreases along the endocytic pathway from values around 6.5 at early endosome to pH ~ 6.5–6.0 at medial endosome to pH around 5.5–4.8 at late endosome and lysosome where lipid processing and loading onto CD1 take place [19]. Therefore, a first key point is that the activity of saposins occurs in acidic media. A second essential feature of these proteins concerns their structure. Saposins show a conserved four-helical fold very similar in size and shape to non-specific lipid transfer proteins (nsLTPs) but with only three disulfide bonds instead of the four ones typical of nsLTPs [20,21,22,23]. In this regard, we have recently presented experimental evidence that supports the participation of the major allergen from peach, Pru p 3, an nsLTP harboring a lipidic ligand that has a phytosphingosine tail, in the trafficking and loading of this lipid onto CD1d [24]. This CD1d loading would activate a cascade of events associated to the immune response in the sensitization phase of food allergy and the possible role of Pru p 3 in the trafficking of its lipid ligand and loading onto CD1d was proposed on the basis of structure and size similarities between Pru p 3 and saposins [24].

The evidence on features and activities of saposins summarized above has accumulated from separate studies over the last years. Saposins are an important class of proteins in processes triggering essential immune responses but they are still not understood in detail. With the aim to try to understand them better, we report here a comprehensive in silico study of the four saposins on the sole basis of their experimental structures. Our goal is to shed light into essential features that can be computationally obtained from the structure and that may provide information on their different behavior upon changes in the acidity of the medium. To reach this goal we analyze two main types of molecular features at different pH values: (*i*) electrostatic properties of the four saposins in their known closed and open forms, and (*ii*) dynamical mobility and flexibility of the open forms known for saposins A, B, and C. To obtain (*i*), we computed Poisson-Boltzmann electrostatic potentials and to study (*ii*), molecular dynamics 100-ns simulations in aqueous solvent were performed. Both studies were carried out at pH 7, 5, and 4.5. While the neutral value is representative of physiological pH for reference, the two acidic values are representative of late endosome and lysosome where saposins process lipids. Furthermore, as demonstrated below, it is just at the narrow pH interval between 4.5 and 5 when all the significant changes occur in the four saposins.

## 2. Results

As of January 15th, 2018, fifteen experimental structures were available in the Protein Data Bank (PDB) for human saposins (Table 1). 

Saposin A has three crystal structures: two for the closed conformation at resolutions 2.00 Å (2DOB) and 1.80 Å (4UEX) and one for the open conformation at 1.90 Å resolution (4DDJ) that corresponds to an aggregate with zwitterionic detergent LDAO with which this saposin forms lipoprotein discs [11]. The two crystal structures available for saposin B at resolution 2.20 Å (1N69) and 2.13 Å (4V2O) are open conformations. The first one is a structure in which the phospholipid PEH was modeled from the X-ray electron density [13] while the second one was obtained in a pharmacological study on the function and toxicity of the antimalarial drug chloroquine in which binding to saposin B was demonstrated [27]. Saposin C has both crystal (PDB id. and resolution in Å: 2GTG 2.40, 2QYP 2.45, and 2Z9A 2.50) and NMR (1M12 and 1SN6, both with 20 models) structures that cover both closed and open conformations. All five of the crystal structures of saposin D correspond to closed conformations; their PDB id. and resolution (parentheses, in Å) are the following: 2RB3 (2.10), 2R0R (2.50), 2R1Q (2.50), 3BQP (1.30), and 3BQQ (2.00). The presence of iodinated tyrosine in 2R1Q is due to heavy atom derivatization used to determine all the related structures (2RB3, 2R0R, and 2R1Q) by molecular replacement [15]. In order to get a comprehensive picture of structure-based features and given that the closed form of saposin B and the open form of saposin D are not available, we generated homology models for these two structures as explained in Methods.

### 2.1. Closed and Open Forms of Human Saposins: Structural and Surface Features

**Figure 1:**
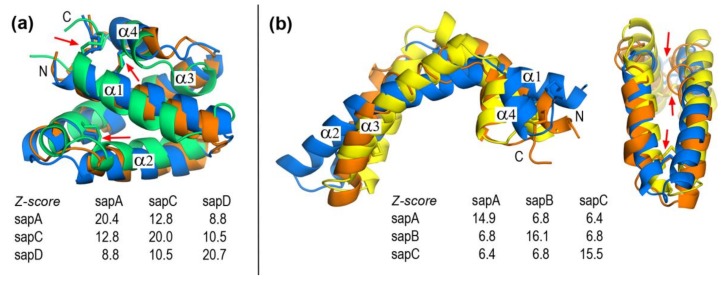
Structural alignment of human saposins obtained with DALI. (**a**) Closed conformations of saposin A (chain A in 4UEX, blue), saposin C (2GTG, orange), and saposin D (chain A in 2RB3, green). (**b**) Open conformations of saposin A (4DDJ, blue), saposin B (chain A in 1N69, yellow), and saposin C (conformation 1 in 1SN6, orange). DALI Z-score matrices of pairwise structural similarities are also shown. Red arrows indicate three conserved interhelical disulfide bridges.

DALI Z-scores (Figure 1) for multiple structural alignments reveal that closed forms show a closer structural similarity than open forms. For a set of *N* structures, this method computes the *N* × *N* matrix of pairwise similarities which are quantified by Z-scores. DALI optimizes this score by using several heuristic procedures complementing structural information with sequence comparisons, resorting in both cases to updated databases [30,31]. For this reason, (*i*,*i*) elements in *N* × *N* matrices may have different values as it is the case with *3* × *3* pairwise similarity matrices for closed and open structures in Figure 1. In this regard, the overall higher values for closed forms (Figure 1a) denote greater similarities than those of open forms (Figure 1b). As for structural relationships within saposin types, the similarity between closed conformations decreases in the order (Z-scores in parentheses) A–C (12.8) > C–D (10.5) > A–D (8.8). In contrast, structural similarities between open conformations are nearly identical, with Z-scores in a narrow range 6.4–6.8 (Figure 1b). This indicates that the closed form of saposins allows for a slightly greater structural variability than open forms that have a rather similar arrangement.

Saposins share less than 40% sequence similarity, although there is a clear difference between saposin B and the remaining saposins. While saposins A, C, and D have pairwise sequence identities ranging from 34.2% to 39.7%, saposin B has lower sequence similarity relative to the other three saposins with pairwise identities ranging from 16.0% to 23.1% (Table 2). Since both saposins B and D show their greatest similarity with saposin A, we selected the closed and open forms of this member as templates to obtain homology-modeled structures of the closed form of saposin B and open form of saposin D that are not present in the PDB (Table 1). In both cases, the modeled geometries are closely similar to their templates (Figure S1) although, as expected from their corresponding sequence identities, the saposin B model shows a slightly greater difference (backbone RMSD = 0.716 Å) than the saposin D model (backbone RMSD = 0.293 Å).

**Figure 2:**
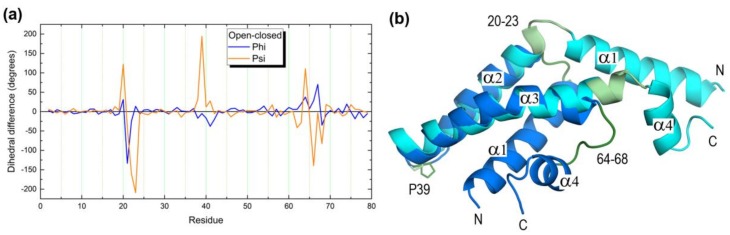
Structural comparison of closed (2DOB, blue) and open (4DDJ, cyan) conformations of human saposin A. (**a**) Plot of (open-closed) difference of backbone dihedral angles φ and Ψ. (**b**) Superposition of closed and open conformations. Segments showing large differences in plot (**a**), 20-23 and 64–68 together with Pro39, are colored in light green (open) and deep green (closed).

Despite their overall low sequence similarities, the four saposins in their closed monomeric form share a similar fold consisting of four α helices arranged in two layers α1/α2 and α3/α4 linked by three interhelical disulfide bridges formed by six conserved cysteines (Figure 1). Two of these disulfide bonds connect N- and C-terminal regions: one -SS- bond links helices α1 andα4 and the other bond links helix α1 and the C-terminal coil segment. The third disulfide bridge connects helices α2 and α3. Linkages between α1 and α2 and between α3 and α4 are short loops that provide hinge-like flexibility (Figure 1). It is worth noting that the three -SS- bridges remain unchanged upon the close ® open conformational change. In fact, the available structures of open forms (saposin A [11], saposin B [13,27], and saposin C [15,29]) show that the closed fold opens in a jackknife style. A comparison between closed and open forms of saposin A (Figure 2) reveals that the structural rearrangement involves residues 20–23 and 64–68 that form the two hinge regions together with small segments of neighbor helices α2 and α3 (Figure 2). The major structural change arises from variations in backbone dihedral angle Ψ (N-Cα-C-N) of residues (open-closed differences > 100° in parentheses): 20 (+122°), 22 (−161°), 23 (−209°), 39 (+194°), 64 (+110°), and 66 (−139°) whereas dihedral angle φ (C-Cα-N-C) shows a variation larger than 100° only in residue 21 (−134°). All these amino acids are located in hinge regions (Figure 2) with the single exception of residue 39, a proline in which the large variation of Ψ corresponds to its different backbone geometry, cis in the closed form and trans in the open form (Figure S2).

**Figure 3:**
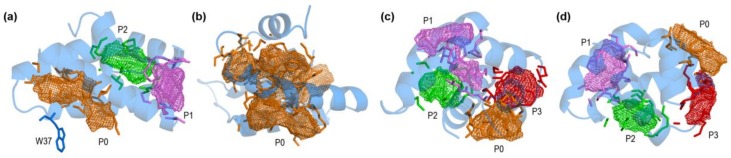
Surface pockets detected by DogSite in the closed conformations of human saposins. (**a**) Saposin A (chain A in 4UEX). (**b**) Modeled structure of saposin B (**c**) Saposin C (2GTG). (**d**) Saposin D (chain A in 2RB3). Mesh isosurfaces represent the pockets and their closer residues are depicted as sticks in the same color. Protruding W37 in saposin A (residue with an important role in the activity of this saposin: see below) is also drawn. Pocket numbering indicates their arrangement in order of decreasing volume (see Table S1).

Although one might expect that α-helical folds involved in transport and processing of lipids should have cavities (of the type nsLTPs have) able to harbor them [20], closed conformations of human saposins exhibit no clear candidates for this. Open conformations are known to aggregate exposing their hydrophobic side to lipids protecting them from the aqueous environment by forming lipoprotein-like particles (see below). This way, these open structures use no internal cavities in the monomers to harbor ligands. However, the possible abilities of closed conformations to transport lipids have been not addressed before. An exploration of surfaces with DogSite [34,35] in the closed forms of saposins searching for pockets potentially able to harbor ligands reveals that the experimental structures of these proteins lack an internal cavity large enough to accommodate molecules such as most of the lipids loaded onto CD1 receptors (Figure 3). Only saposin C has an inner small compartment that connects to the outer larger domain of a pocket (P1 in Figure 3b) whereas in all the remaining cases, surface sites candidate to ligand-binding sites are invariably located at the external surface (Figure 3). Moreover, as the nature of the residues in these pockets reveals (see Table S1) and is further supported by the electrostatic features of molecular surfaces (see below), these sites are enriched in polar and charged residues. This way, although the topography and size of local surface sites lead to identify candidate sites for ligand binding [34], they do not seem particularly suited for accommodating lipids. A druggability score to estimate the probability that an identified pocket could harbor ligands, evaluated by DogSite in terms of site descriptors that also treat hydrophobicity [35], predicts relatively low binding trends for all these pockets. Values of this score defined in a (0–1) scale are lower than 0.50 (Table S1) and only in saposin A they reach values greater than 0.40 (pockets P0 and P1 in Figure 3a). Tryptophan 37, which is unusually protruding and plays an important role in the activity of saposin A (see below) is close to pocket P0 but its side chain is oriented outwards (Figure 3a). A supplementary exploration of all experimental closed forms of saposins in Table 1 with a different method to detect cavities such as that implemented in the Swiss-PdbViewer software [36] was also unable to find any inner cavity (results not shown). It seems apparent that these closed forms do not provide favorable hydrophobic inner environments for harboring lipidic ligands. 

Interestingly, the modeled closed conformation of saposin B exhibits a large internal cavity which extends all the way through the structure (Figure 3b). Its volume, number of residues, and high druggability 0.85 (Table S1) indicate that this cavity should be able to harbor large ligands. However, since experimental structures of saposins are obtained from biological samples and this is the only missing closed form, one might speculate that saposin B never adopts a closed conformation in its actual environments in cells. This should indeed be consistent with our dynamical study of the open conformation of saposin B that, as shown in 2.3 below, reveals a particular stability at all pH values which in turn agree with the supposed mechanism of its function [13,27]. In fact, the evidence on saposin B indicates that a dimer formed by two clasping monomers extracts lipids from membranes accommodating them in the hydrophobic pocket formed by the clasping chains [13].

Another structural feature is the existence of alternative conformations of several side chains identified by fractional values of occupancy in the electron density in some crystal structures (Table S2). Although most of these residues show equivalent conformations (occupancy factors 0.50), some amino acids in closed saposin A at lysosomal pH [26] have occupancies favoring one conformation with values between 0.51 and 0.66 (Table S2). With a unique exception (Met66 in chain A), all these residues are in outside hinge regions and all except two are polar or charged amino acids (Figure S3). The two nonpolar exceptions are Ile50 that in chain B shows two very similar conformations oriented inwards, and Trp37 which is dramatically exposed to solvent (Figure 3a), locates at the loop that joins helices α2 and α3, and shows two distinct orientations (Figure S3). As discussed below, this residue is believed to play a prominent role in the activity of saposin A. In fact, experimental studies of the interaction between this saposin and phosphatidylserine liposomes using tryptophan fluorescence spectroscopy and quenching measurements showed that Trp37 can associate with lipids and insert into membranes [37]. Recent tryptophan fluorescence spectroscopy monitoring amphiphile-induced spectral shifts also revealed that Trp37 is associated to lipids, regardless of their composition [11]. Trp37 together with Tyr30 (also solvent-exposed and located in the α2 helix) have mobile side chains and both are thought to facilitate association of closed saposin A with membranes prior to lipid binding and lipoprotein assembly formation [26]. Tyr30 is conserved in saposin A orthologues and Trp37 is not fully conserved but it is substituted by other hydrophobic amino acids such as Phe or Leu, supporting a possible functional role through membrane insertion of their bulky hydrophobic side chains. It must be stressed, though, that Ty30 and Trp37 are not conserved among different saposins, which suggests for them a role in lipid interactions that differs between saposins [26].

### 2.2. Closed and Open Forms of Human Saposins: Electrostatic Features

All human saposins are acidic proteins, with total electric charges at physiological pH ranging from −4 in dimeric closed forms of saposin D to −9 in the monomeric closed form of saposin C (Table 3). These charges arise from the balance between a greater number of acidic residues (15 in saposin A, 11 in saposin B, 16 in saposin C, and 12–13 in saposin D) than basic residues (7 in saposin A, 5 in saposin B, and 8 in saposins C and D) (Table 3). 

**Figure 4:**
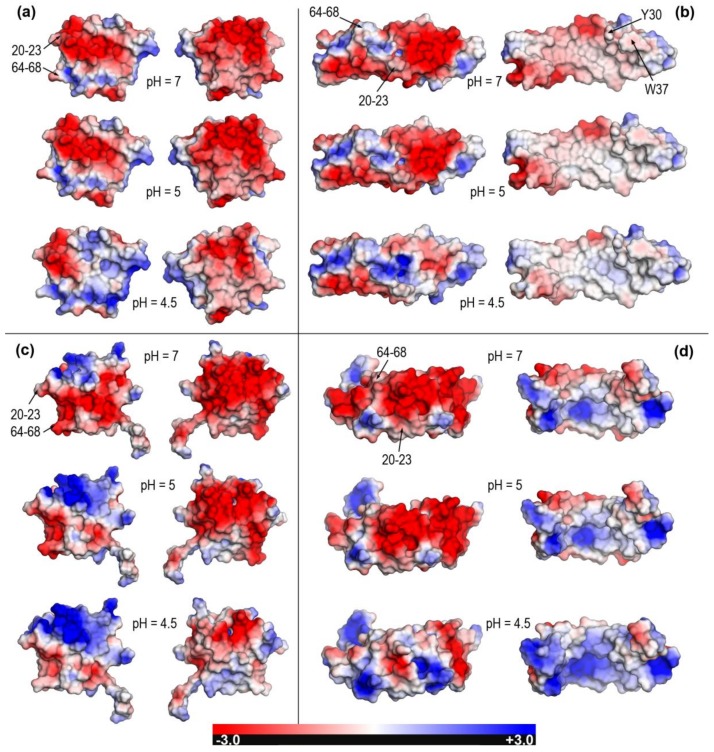
Poisson-Boltzmann electrostatic potential mapped onto molecular surfaces of saposins A and C at pH 7, 5, and 4.5. (**a**) Closed form of saposin A (chain A in 4UEX). (**b**) Open form of saposin A (4DDJ). Surfaces of Y30 and W37 residues with solvent-exposed protruding side chains are indicated only in the first row. (**c**) Closed form of saposin C (conformation 1 in 1M12). (**d**) Open form of saposin C (conformation 1 in 1SN6). Right views in each panel are obtained from left views upon 180^o^ rotation around a vertical axis. Segments involved in the closed → open transformation (Figure 2) are labeled only in left images of first rows.

**Figure 5:**
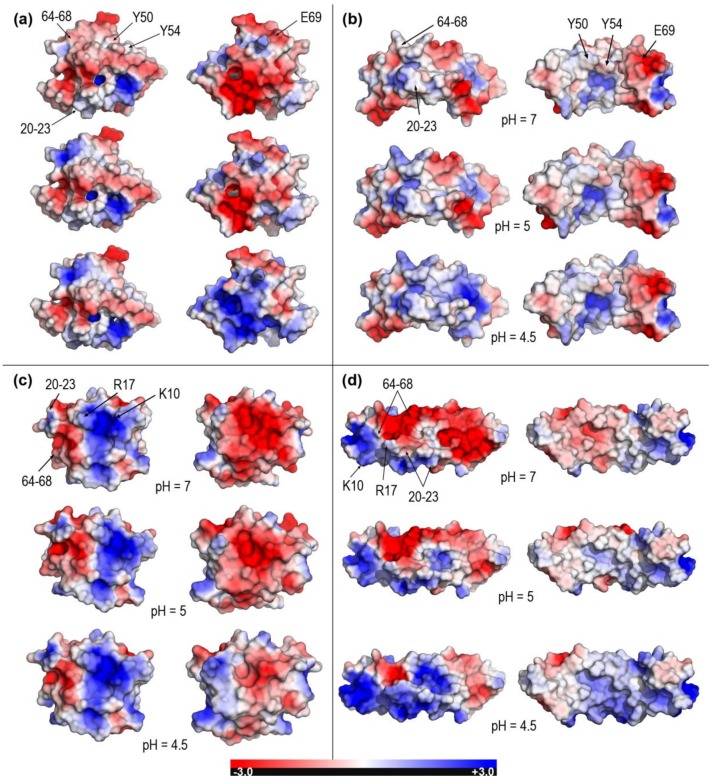
Poisson-Boltzmann electrostatic potential mapped onto molecular surfaces of saposins B and D at pH 7, 5, and 4.5. (**a**) Modeled closed form of saposin B. (**b**) Open form of saposin B (chain A in X-ray structure 1N69). Surfaces of Y50, Y54, and E69 residues involved in drug- and lipid-binding are indicated only in the first row. (**c**) Closed form of saposin D (chain A in X-ray structure 2RB3). (**d**) Modeled form of saposin D. Surfaces of K10 and R17 residues involved in association with anionic headgroups of lipids in membranes are indicated only in the first row. Right views in each panel are obtained from left views upon 180° rotation around a vertical axis. Segments involved in the closed → open transformation (Figure 2) are labeled only in left images of first rows.

However, acidic pH values in the narrow interval 5–4.5, representative of environments where lipid processing and loading onto CD1 occur [19], force changes in the protonation state of ionizable side chains which in turn leads to significant variations of electric charge that differ largely between saposins. In the absence of sufficient experimental data to address all the pH-dependent changes in sufficient detail for all the structures, we resorted to theoretical estimates of pKa’s in order to present a comprehensive comparative analysis of the four saposins, including the two structures modeled in this work. The empirical method used, Propka 3.1 [38,39], has shown a reasonable reliability in a variety of proteins at both internal and surface structural regions [38]. For the particular case of saposins and taking the scarce experimental data available for comparison as reference, our results in Table 3 agree with measured total charges of saposin A (2DOB) at pH 7 (−8.2) and pH 4.8 (~−3) and saposin C (2GTG) at pH 7 (−9.1) [25]. In agreement with that observed in the NMR structure of closed saposin C (1M12 [28]), our computed pKa values (data not shown) also agree in predicting anomalously high values for its exposed glutamates. As an overall result, whereas saposin A maintains its negative charge at low pH, the remaining saposins show zero or even positive charge at pH 4.5 (Table 3). All these variations reflect into the electrostatic nature of molecular surfaces that also changes dramatically at pH between 5 and 4.5 as revealed by Poisson-Boltzmann (PB) electrostatic potentials (EPs) mapped onto surfaces (Figure 4 and Figure 5).

Electrostatic changes are all the more significant when closed and open forms of the same saposin can be compared as it happens in experimental structures for saposins A and C (Figure 4) and for both experimental and modeled structures for saposins B and D (Figure 5). The closed form of saposins A and C shows a strong EP in both sides of the surface although one side is predominantly negative (right column in Figure 4a,c) and the other side has large patches of positive PB-EP (left column in Figure 4a,c). In both cases, however, an increasingly acidic medium provokes a decrease in intensity and extension of surface areas with negative potential in the negative side and an increase in intensity and extension of surface areas with positive potential in the positive side (Figure 4a,c). On the contrary, the open conformation shows a dramatic difference between one side with large areas of intense both negative and positive PB-EP (left column in Figure 4b,d) and the other side with a PB- EP predominantly neutral in saposin A (right column in Figure 4b) and predominantly positive in saposin C (right column in Figure 4d). Again, lower pH provokes a decrease in the extension of surface areas with negative EP and an increase in intensity and extension of areas with positive EP (Figure 4b,d) yet with a significant difference with regard to saposin A in which the electrostatically neutral side changes very little at lower pH (right column in Figure 4b). These electrostatic features are consistent with the two distinct mechanisms proposed for lipid loading by saposins. As said in the Introduction, saposin A should form protein-lipid soluble aggregates with lipids extracted from membranes whereas saposin C should embed into the membrane disrupting the bilayer. In the first case, saposin A would employ its nonpolar, electrostatically neutral side to form aggregates with lipids arranged within an inner hydrophobic enclosure and, as mentioned above, the solvent-exposed protruding hydrophobic side chains of Tyr30 and Trp37 in this nonpolar side (Figure 4b) could also be inserted around headgroups of phospholipids into membranes [8,9,26]. In the second case, saposin C would use its large surface patches with positive EP as putative contact sites with membranes.

Figure 5 displays PB-EPs mapped onto molecular surfaces of saposins B and D for which only open [13,27] and closed [15,17] forms, respectively, exist in their experimental structures. For a comprehensive analysis, modeled structures of the closed form of saposin B and open form of saposin D are also included. Saposin B presents two sides with electrostatic differences smaller than other saposins. As pH decreases, one side increases significantly the intensity and extension of patches with positive PB-EP (right column in Figure 5a and left column in Figure 5b) whereas the other side, which has a large patch with neutral PB-EP that covers most of the surface, displays nearly insignificant changes (left column in Figure 5a and right column in Figure 5b). The first crystal structure reported for saposin B (1N69) contains in the asymmetric unit three independent chains in a V-shape open form that gives rise to two distinct homodimers [13]. This structure helped explain how such a small protein could solubilize large lipids. In the dimeric arrangement, two concave inner surfaces of the “V” with large electrostatically neutral patches (right column in Figure 5b) clasp together enclosing a hydrophobic cavity in which two tyrosines (Tyr50 and Tyr54) have very protruding side chains, an identical feature to that observed for Trp37 in saposin A above mentioned. When the 1N69 structure was reported in 2003, it was noted that this cavity had a large solvent-exposed region at which polar headgroups of lipids might locate [13]. In fact, the PB-EP in Figure 5b supports this view. The arrangement of two concave surfaces must leave a large part showing a negative PB-EP due to the presence of a largely exposed Glu69 (rightmost sides in the right column in Figure 5b). The other crystal structure of saposin B (4V2O) reported in 2016 is a complex of the dimeric state with the drug chloroquine [27] although the drug-binding site is now exposed to the surface and corresponds to the region with negative PB-EP around Glu69, the most relevant residue in binding to chloroquine [27]. Whereas no information is available for pH-dependent changes of the solubilizing action of saposin B on large lipids [13], chloroquine binding to this saposin was found to be very similar at pH 7.4 (K_D_ = 31.8 µM) and pH 5.5 (K_D_ = 40.8 µM) [27]. This result agrees with our above comment regarding the smaller electrostatic differences noticed in saposin B with respect to other saposins. As for the closed form of saposin B, the available evidence [13,27] suggests that it is highly unlikely that it could function as a monomer. Therefore, whereas the electrostatic features of the model closed structure are compatible with the remaining PB-EP results, they are scarcely significant.

Saposin D, for which only closed forms exist in their crystal structures [15,17], presents now clear electrostatic differences between one side with dominantly positive PB-EP (left column in Figure 5c) and another side with dominantly negative PB-EP (right column in Figure 5c). These marked differences are also found in the modeled structure of its open form (Figure 5d). Whereas increasingly acidic pH provokes a clear decrease in intensity and extension of negative areas in the negative side and appearance of surface regions with positive potential at pH 4.5 (right column in Figure 5c and left column in Figure 5d), the effects on the positive side (left column in Figure 5c and right column in Figure 5d) are barely noticeable. According to the model of lipid activation proposed for saposin D, water-soluble monomers and possibly dimers of this saposin would bind to negatively charged membrane surfaces [15]. Two residues, Lys10 and Arg17, would particularly be involved in the initial association of saposin D with anionic membrane regions by forming ionic pairs with sulfate or phosphate headgroups [15]. The information provided by our PB-EP results (Figure 5c,d) supports these proposals. In fact, the surfaces of Lys10 and Arg17 are predominant in a large elongated patch with positive PB-EP which should arrange linearly along the membrane length (see Figure 4 in [15] for a proof of consistency between this proposal and Figure 5c). While this saposin D-membrane interaction should hold at the three pH values studied here, our results suggest a favored association to membrane anionic regions at increasingly acidic pH, reinforced by the enlarged surface areas with positive PB-EP at pH 4.5. The fact that saposin D in two different crystal forms at distinct pH (orthorhombic obtained at pH 8.5 and triclinic at pH 4.8) is monomeric in solution suggests that this saposin may have a weaker propensity for dimerization and a lesser pH-dependence than other saposins [17]. If the saposin D-membrane interaction were to involve only monomers and Lys10 and Arg17 are the main responsible for the association, our PB-EP results on the modeled open structure (Figure 5d) indicate that the outer side of the open conformation facing the aqueous environment when the saposin is located at the membrane would be mostly non-polar except at the lowest pH 4.5 (right column in Figure 5d). From a purely electrostatic point of view, this result seems to preclude the open form of saposin D in its proposed role [15,17].

### 2.3. Open Forms of Human Saposins: Dynamic Features

**Figure 6:**
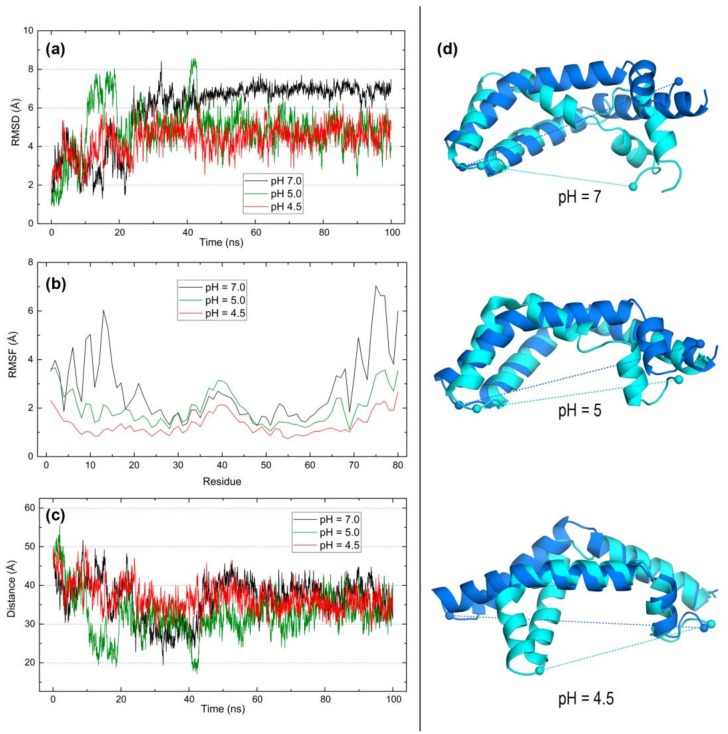
Results of 100-ns MD simulations on the open form of saposin A (4DDJ) at pH 7, 5, and 4.5. (**a**) RMSD of backbone atoms. (**b**) RMSF of all residues. (**c**) Distance between alpha carbons of P39 and E80 residues. (**d**) Structural superposition of initial (blue) and final (cyan) structures in the simulations computed with TM-Align [42]. Dashed lines indicate distances between Cα atoms of P39 and E80 represented as spheres.

**Figure 7:**
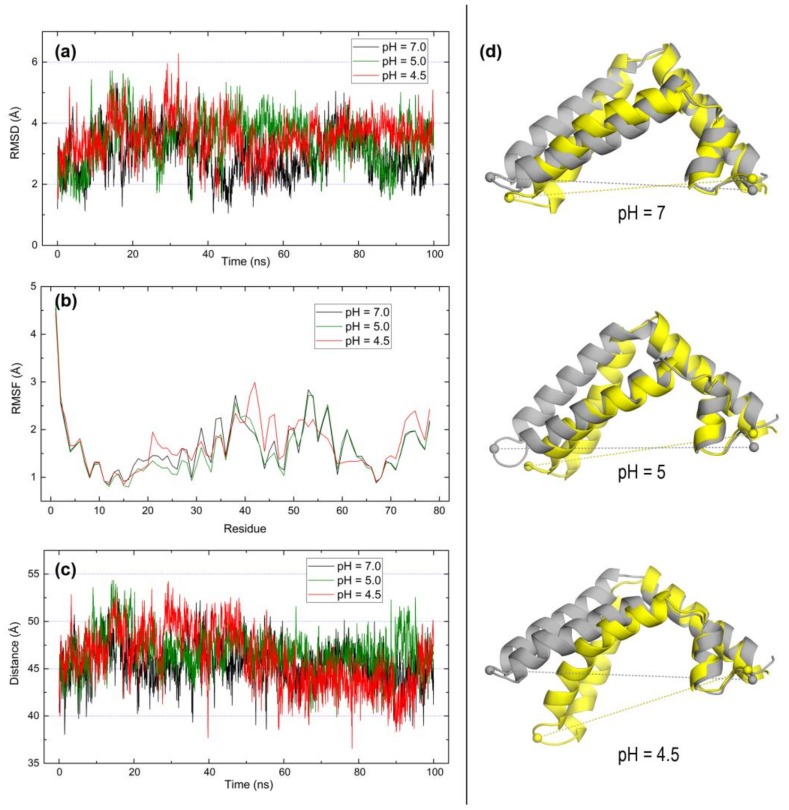
Results of 100-ns MD simulations on the open form of saposin B (chain A in 1N69) at pH 7, 5, and 4.5. (**a**) RMSD of backbone atoms. (**b**) RMSF of all residues. (**c**) Distance between alpha carbons of P41 and D78 residues. (**d**) Structural superposition of initial (yellow) and final (grey) structures in the simulations computed with TM-Align [42]. Dashed lines indicate distances between Cα atoms of P41 and D78 represented as spheres.

**Figure 8:**
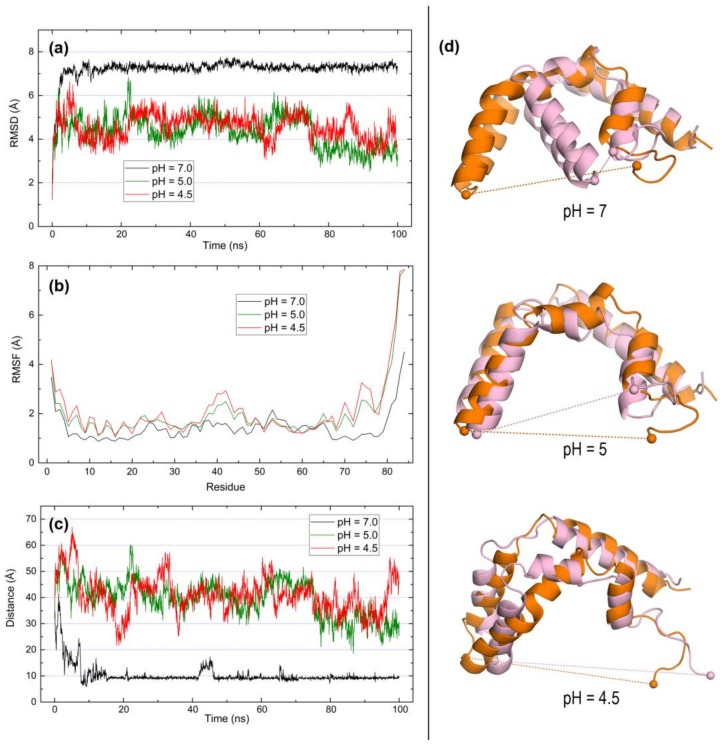
Results of 100-ns MD simulations on the open form of saposin C (chain 1 in 1SN6) at pH 7, 5, and 4.5. (**a**) RMSD of backbone atoms. (**b**) RMSF of all residues. (**c**) Distance between alpha carbons of P40 and R84 residues. (**d**) Structural superposition of initial (orange) and final (pink) structures in the simulations computed with TM-Align [42]. Dashed lines indicate distances between Cα atoms of P40 and R84 represented as spheres.

Results of 100-ns MD simulations for open forms of saposins A, B, and C are presented in Figure 6, Figure 7 and Figure 8, respectively. The experimental structures chosen to address dynamic features of the open conformations were the following: 4DDJ, the unique open structure of saposin A; 1N69, the only structure of saposin B obtained in complex with a membrane lipid (the other available structure corresponds to a saposin-drug complex); and 1SN6, the unique monomeric open structure of saposin C. The following data are presented: (a) variation along simulation time of root mean square deviation (RMSD) of backbone atoms with respect to the initial structure, (b) root mean square fluctuations (RMSF) of α carbons of all residues with respect to time-averaged positions, (c) variation along simulation time of the distance between α carbons of residues located at opposite ends of the backbone, and (d) structural superposition of initial and final structures in the simulation computed with FATCAT rigid [40], CE [41], and TM-Align [42] structural alignment methods. Significance scores for these initial/final structural comparisons are gathered in Table 4. To assess these scores, let us recall that (*i*) FATCAT rigid P-values lower than 0.001 are considered to indicate structural relationship between the structures being compared, with lower values indicating higher similarity [40], (*ii*) CE Z-scores between 3.0 and 4.0 suggest some structural similarity, with values increasingly greater than 4.0 revealing stronger structural resemblance [41], and (*iii*) TM-Align TM-scores in the scale (0.0–1.0) indicate higher similarity as values get closer to 1.0 [42].

The RMSD plot for open saposin A (Figure 6a) reveals a steady mobility after about 40 ns regardless of the pH although the backbone is less mobile at acidic pH than at neutral pH, with no significant differences between pH 5 and 4.5. The RMSF plot (Figure 6b) shows that only ~1–20 and ~70–80 terminal segments fluctuate to a much larger extent than the remaining residues at neutral pH whereas acidic environments stabilize the whole sequence. Segments 20–23 and 64–68 found to be responsible for the closed → open transformation in this saposin (Figure 2) not only have low fluctuations but they also change little with pH. Interestingly, a larger fluctuation is found at the three pH values for the central segment around position 40. This segment corresponds to the loop joining α2 and α3 helices and contains Pro39, residue chosen to measure the distance between opposite ends. RMSF plot indicates that increasingly acidic environments stabilize fluctuations in the whole protein with a marked effect at the lowest pH 4.5 (Figure 6b). As for the larger mobility associated to both N- and C-term ends, the Pro39-Glu80 distance (Figure 6c) also stabilizes at a value ~35 Å after about 40 ns, just like that found for RMSDs. The comparison between initial and final structures (Figure 6d) reveals that the chain bends keeping unaltered the helices held by disulfide links and that the final distance between opposite ends decreases noticeably from ~50 Å at the three pHs. This suggests that the dynamic behavior of this open form in the absence of lipids leads saposin A to partially close, thus decreasing its degree of opening. Structural comparisons of initial and final geometries in saposin A at the three pHs reveal differences greater than those found in the other saposins, as shown in Table 4 and discussed next.

In fact, MD data for saposin B provide a rather distinct picture with scarce differences at the three pH values. RMSD (Figure 7a), RMSF (Figure 7b), and distance (Figure 7c) plots show remarkably similar patterns irrespective of pH. Backbone mobility indicated by average RMSD values below 4 Å is lower than in saposin A, no RMSF greater than 3 Å is noticed in the whole protein with the single exception of the N-terminal residue, and the distance between Pro41 and Asp78 at opposite ends of the structure deviate little from an initial value around 45 Å. As indicated by both the structural superposition (Figure 7d) and scores in the three structural alignment methods (Table 4), saposin B seems to have a steady dynamic behavior which is practically unaffected by pH variations. This is in agreement with the supposed function of this saposin B in extirpating lipids from membranes adopting a dimeric state formed by two clasping monomers in open conformation at acidic pH [13,27].

Finally, saposin C exhibits great differences not only in its distinct behavior at neutral and acidic pH but also with respect to both saposins A and B. The RMSD plot (Figure 8a) is a nearly flat line at pH 7 indicative of small backbone mobility, with a large value about 7 Å which is reached almost immediately after starting the simulations. No significant differences between the open form at pH 5 and 4.5 are noticed and the mobility is not only lower than in saposins A and B but it decreases slightly with simulation time and at 80 ns, the RMSD is ~4 Å. As for residue fluctuations, the RMSF plot (Figure 8b) suggests a behavior similar to saposin A although RMSF values are now greater at pH 4.5 than at pH 7 whereas just the opposite was observed in saposin A (Figure 6b). At the two acidic pH values, the C-terminal segment (positions ≥ 70) fluctuates to a much greater extent than the remaining chain which, according to the structural superposition (Figure 8d) might be due to the flexibility of the C-term coil segment. The distance plot (Figure 8c) is consistent with that view. The distance between Pro40 and Arg84 at opposite ends increases from an initial value ~40 Å and then oscillates around that value with deviations above 10 Å at both pH 5 and 4.5. However, at pH 7 this distance falls rapidly and at ~10 ns it stabilizes at a nearly unchanged value ~10 Å that corresponds to the geometry displayed in the top view of Figure 8d: the open chain has closed tightly and the initially separated Pro40 and Arg84 residues are then in close proximity. The information provided by structural superposition scores (Table 4) reinforces this view: at pH 7 the similarity between initial and final geometries is low (FATCAT P-value is the greatest and CE Z-score is the smallest being the unique value of this parameter below 4.0 in the table) whereas at both pH 5 and 4.5 the scores reveal that the similarity increases significantly. Summarizing, the dynamic behavior of saposin C indicates that, in the absence of lipids, this protein seems to need an acidic environment to keep its open form. Our MD results are thus consistent with available data suggesting that saposin C embeds into membrane at endosomal acidic pH disrupting the bilayer lattice and orienting CD1 receptors towards accessible lipids while it remains in aggregation states formed by the open conformation [5,6,8].

## 3. Discussion

Saposins share with other small proteins involved in lipid transport such as nonspecific lipid transfer proteins (nsLTPs), a fold consisting of four α-helices linked by disulfide bonds. By using helical layering, this architecture allows them to form modular compartments enriched in non-polar side chains for lipid-binding [20]. Together with this hydrophobic environment, these proteins must also be able to interact with polar headgroups present in a large variety of lipids. For this, lipid-binding proteins usually have a number of polar and/or charged residues in the proximity of their hydrophobic sites. However, in spite of this structural similarity to nsLTPs, our study indicates that saposins apparently make no use of internal cavities to harbor and transport lipids, a result consistent with the available evidence on their lipid-binding features.

Scattered information on the lipid-binding abilities of saposins gives clues about their specific properties that may be grouped in two categories: pH-dependence of interactions with lipids and existence of closed/open alternative forms of their helical structure. As early as in 1995, experimental evidence provided by phase partitioning in Triton X-114 demonstrated that saposins A, C, and D acquired hydrophobic properties upon acidification, while saposin B was apparently unaltered [43]. Over the last 15 years, it has been repeatedly proposed that lipid-binding in saposins is pH-dependent [11,15,17,25,26,27,28,29,44], a property which calls for pH-controlled reversible neutralization of residues with ionizable side chains. Saposins work in lysosomes, where the medium is maintained at pH values in the range 4–5.5 [43,44] but pH alone does not suffice to explain the closed → open conformational change [11,25,26].

Saposin A remains closed at lysosomal pH 4.8 [26] and only in the presence of lipids or detergents, it undergoes the structural change to its open conformation forming lipo-protein particles with a variety of lipids [11,25,26]. Similar behavior on pH- and detergent-induced oligomerization is observed for saposin C, probably the best studied saposin [25]. In the absence of lipids, saposin C adopts the closed, compact form that buries hydrophobic residues, whereas in the presence of SDS micelles, it adopts an open V-shaped conformation [29]. pH titration measurements on saposin C revealed that about 50% of its glutamates were neutralized at pH ~ 5 although no conformational change occurred when the pH was lowered from pH 7 to 5 [29]. However, neutralization of acidic residues in this saposin was thought to facilitate interaction with membranes as the negatively charged surfaces might cause electrostatic repulsion from negatively charged groups of the membrane [28,29]. In this regard, it has also been proposed that several lysines in saposin C might contribute to interaction with membranes [29]. As for oligomer states, saposin C has been reported to be dimeric [15,44] or trimeric [25] in solution at low pH. The suggestion that dimers shield the hydrophobic surface sides of open monomers agrees with that observed in the crystal structure of open saposin A in complex with detergent lauryldimethylamine-N-oxide (LDAO) that reveals two open chains encapsulating 40 internally bound LDAO molecules [11].

Saposin B has been reported as the dominant saposin in facilitating lipid binding to CD1d molecules [12]. Although all saposins promote lipid binding to CD1d, it seems that saposin B is the most efficient and its optimal pH is 6, higher than that of lysosomes, which suggests that saposin B should facilitate lipid binding to CD1d throughout the endocytic pathway [12]. While the affinity for phospholipid membranes of saposins A, C, and D depends on low pH, that of saposin B does not [12,43]. Furthermore, in contrast with initial reports suggesting that saposin B had weak affinity for phospholipids [12], more recent work indicates that it binds, transports and transfers a large variety of membrane sphingolipids and phospholipids as well [45]. In any event, it seems that saposin B functions as a lipid extractor and solubilizer that interacts only transiently with membranes. In fact, this saposin extracts target lipids from membranes and forms soluble protein-lipid complexes in its dimeric state formed by the open conformation [13]. While saposins A and C require the presence of lipid/detergents for dimerization, saposin B is dimeric in the presence or absence of detergents at both neutral and low pH [25]. This dimer is formed with two V-shaped open clasping monomers and binds lipids in a large hydrophobic compartment with an opening in which lipid polar headgroups remain exposed to solvent [13]. It has also been proposed that this internal cavity can contain more than one lipid molecule [45].

Saposin D is a ceramide activator protein required to activate the hydrolysis of ceramide to a fatty acid and sphingosine by acid ceramidase [46,47]. Saposin D, contrarily to that observed in other saposins, shows in its crystal structures a compact closed monomeric form at basic, neutral, and acidic pH [15,17]. Nevertheless, this does not exclude the possibility that weak interactions occurring in vivo could form a saposin D dimer [17]. Lipid binding and sphingolipid activation function of saposin D requires acidic pH and the presence of anionic phospholipids [17,47,48].

Our compared study of saposins sheds new light upon the pH-dependence of key features of these proteins. Thus, data reported here on electrostatic potentials mapped onto molecular surfaces reveal the precise location of regions especially sensitive to changes in protonation states of ionizable residues in the narrow pH interval between 4.5 and 5. With the single exception of saposin B, saposins exhibit significant electrostatic changes at increasingly acidic environments. This is particularly noticeable in the finding that large surface regions with strongly negative potential vanish and concomitantly large surface regions with strongly positive potential appear at pH 4.5. This result is observed in both closed and open forms with the exception now of the open conformation of saposin A that shows a nearly neutral (non-polar) side which is the hydrophobic surface that aggregates to lipids/detergents when forming lipo-protein particles and that is found here to remain largely unaltered upon acidification. Our analysis also provides the precise location as well as the pH-dependent changes of particular residues that have been proposed to play prominent roles in saposin interactions (e.g., Tyr30 and Trp37 in saposin A, Tyr50 and Tyr54 in saposin B, or Lys10 and Arg17 in saposin D). Furthermore, since it has been argued that lysine-rich surface patches could be possible contact sites with membranes in all saposins, our results allow for a detailed location of such regions with strongly positive electrostatic potential. As mentioned above with regard to saposin C, it is assumed that these positively charged clusters would interact with negatively charged lipid headgroups in saposin-membrane interactions.

Our study on protein surfaces indicates that closed forms of saposins lack internal cavities that could accommodate lipid molecules. In fact, no inner cavities are detected in experimental structures of closed forms of saposins whereas putative lipid-binding sites are predicted to locate on their outer surface. Moreover, an internal cavity able to harbor ligands is only predicted just for the modeled structure of the closed conformation of saposin B which, as repeatedly indicated above, plays its lipid-extractor role through a dimeric aggregation of its open conformation. The absence of an internal cavity to carry ligands in the helical monomeric fold is at odds with nsLTPs, proteins very similar in structure and size also involved in lipid binding that transport their cargo at an inner tunnel-like cavity lined with hydrophobic amino acids whereas they have polar/charged amino acids at the tunnel entry that interact with headgroups of lipids [20,22]. Recent results demonstrating the presence of the natural ligand carried by Pru p 3 (an nsLTP which is the major allergen from peach fruit) loaded into antigen-presenting CD1d in the sensitization phase of food allergy, suggests the participation of nsLTPs in lipid loading and trafficking in the presentation of antigens to T cells [24]. Given the great structural similarity between Pru p 3 and the closed forms of saposins, one could think of a role played by nsLTPs that are able to trigger immune responses (such as allergens) in lipid processes in which saposins are known to be involved. 

Finally, the current report presents novel results on the pH-dependent dynamic behavior of available open forms of saposins. The results of our molecular dynamics simulations at pH 7, 5, and 4.5 reveal distinct dynamic features in the three saposins for which experimental open structures are available. In saposin A, the backbone mobility as well as the overall fluctuations of all its residues are noticeably greater at neutral pH and decrease at low pH but no tendency to close is observed at any pH. In saposin B, no significant differences are found neither in backbone mobility nor in residue fluctuations nor in the tendency to close at the three pH values considered in our study. In sharp contrast, saposin C exhibits much smaller backbone mobility at low pH than at neutral pH at which the structure deviates largely from the initial geometry. Residue fluctuations are now greater at pH 5 and 4.5 than at pH 7 and this saposin exhibits a strong tendency to close at neutral pH whereas it remains open at acidic pH. This result suggests that this saposin is the most sensitive to acidification and/or to the presence of lipids in order to preserve its open conformation.

## 4. Materials and Methods 

### 4.1. Structural Analyses

Structure for both closed and open forms are available for saposins A and C whereas saposin B has only open and saposin D only closed structures in the PDB (Table 1). The following PDB entries were used for structural comparison analyses: 4UEX in saposin A, 2GTG in saposin C, and 2RB3 in saposin D for the closed forms, and 4DDJ in saposin A, 1N69 in saposin B, and 1SN6 in saposin C for the open forms. Multiple structural alignments were obtained using the http://ekhidna2.biocenter.helsinki.fi/dali/ server [30] that implements DALI [31]. In this method, structural relationships among *N* proteins are evaluated in terms of the *N* × *N* matrix of pairwise similarities (DALI Z-scores) [30,31].

To analyze the closed → open structural change, we selected the structures 2DOB (closed) and 4DDJ (open) of saposin A and compared their backbone dihedral angles φ and Ψ. To avoid possible structural bias due to pH effects and given that two crystal structures are available for the closed conformation of this saposin, we selected 2DOB instead of 4UEX because the former was determined at pH 6.0 [25], similar to that of the open conformation 4DDJ obtained at pH 6.5 [11], whereas the latter corresponds to lysosomal pH 4.8 [26]. Multiple sequence alignments for the four saposins were obtained with the Clustal Omega server at the EMBL-EBI (Hinxton, Cambridge, UK: https://www.ebi.ac.uk/Tools/msa/clustalo/ [32,33]).

Surface pockets were detected in the closed conformations of saposins A, C, and D with the DogSite algorithm implemented in the web server http://proteinsplus.zbh.uni-hamburg.de/#dogsite [34,35]. This method applies a Difference of Gaussian filter to detect potential binding pockets in the molecular surface based exclusively upon the structure of the protein. Size, shape, area, and volume are calculated for each identified pocket together with a druggability score computed with a linear combination of pocket descriptors such as volume, hydrophobicity, and enclosure [35]. This score, defined in the range 0–1, can be taken as an estimate of the expected tendency of each pocket to harbor ligands, with higher scores indicating greater tendency. All molecular graphics were prepared and rendered with PyMOL 2.0.3 [49].

### 4.2. Structural Homology Modeling

Structures for closed and open conformations of saposins B and D (both missing from the experimental structures available in the PDB for human saposins) were constructed by homology modeling using the SwissModel server https://swissmodel.expasy.org/ [50,51]. The *User Template Mode* was selected, taking saposin A for reference in both models by providing the following input templates: chain A of 4UEX (X-ray structure of the closed form of saposin A) for the closed model of saposin B and 4DDJ (X-ray structure of the open form of saposin A) for the open model of saposin D.

### 4.3. Calculations of pKa and Protonation States

pKa values of ionizable side chains were computed with Propka 3.1 [38,39]. This is an updated version of the Propka empirical predictor that implements a new algorithm for modeling non-covalently coupled residues which can influence the titration of each other because of their close proximity [39]. Protonation states were obtained at pH 7, 5, and 4.5. Besides neutral pH taken as reference, the two acidic values represent the cellular environment where lipid processing and loading onto CD1 molecules occur at lysosomes and late endosome [19]. As presented in Results, electrostatic features of saposins show significant changes in this small pH range. Protonation states of all residues with ionizable side chains were assigned at each pH with Propka 3.1 implemented in the Pdb2pqr server http://nbcr-222.ucsd.edu/pdb2pqr_2.0.0/ [52,53] which adds hydrogens as needed by each particular protonation state and optimizes local conformations to fix possible steric clashes. The main output of Pdb2pqr/Propka 3.1 is a PQR file: a modified PDB file in which occupancy and B-factor entries are replaced with atomic charges and radii, respectively [52]. For reasons of compatibility with the force field used in molecular dynamics simulations, Pdb2pqr/Propka 3.1 calculations were done selecting CHARMM [54] atomic charges. This way, a set of PQR files with proper protonation states of ionizable side chains at pH 7, 5, and 4.5 was obtained for each saposin. 

### 4.4. Poisson-Boltzmann Electrostatic Potentials

Using those PQR files as input, Poisson-Boltzmann (PB) electrostatic potentials (EPs) were computed solving the PB equation with the Adaptive Poisson Boltzmann Solver (APBS) 1.4.1 program [55,56]. The nonlinear PB equation was solved in sequential focusing multigrid calculations in 3D grids of 97^3^ = 912,673 points (step size ~0.5 Å) at 310 K and 0.150 M ionic concentration. Dielectric constants 4 for proteins and 78.54 for water were used. The numerical output of PB electrostatic potentials was saved in OpenDX scalar data format, mapped onto molecular surfaces and rendered with PyMOL2.0.3 [49]. PB potentials are given in units of *kT* per unit charge, *k* being Boltzmann’s constant and *T*, absolute temperature.

### 4.5. Molecular Dynamics Calculations

Open conformations of saposins A (4DDJ), B (chain A in 1N69), and C (conformation 1 in 1SN6) were subjected to 100-ns molecular dynamics (MD) simulations with protonation states corresponding to pH 7, 5, and 4.5 obtained above. These calculations used the CHARMM 3.6 force field for proteins [54,57] and were performed with the high performance computing Linux-Power-MPI version of NAMD 2.12 [58] in the Magerit supercomputer of the Technical University of Madrid. Proteins were immersed in 3D periodic solvation boxes with 15 Å spacing and water molecules added according to the TIP3P model [59]. Na^+^ and Cl^−^ ions were added to counter total charges of proteins while providing 0.150 M salt concentration. The particle-mesh Ewald summation method [60] was used for long-range electrostatics and a 10 Å cutoff was set for short-range non-bonded interactions. For every saposin structure (1) the initial geometry was optimized at 2000 conjugate-gradient minimization steps, (2) water was equilibrated at 298 K and 1 atm for 100 ps at 2 fs time steps, and (3) 100-ns simulations at 2 fs time steps (50 million steps per calculation) were run in NPT ensemble at 298 K and 1 atm. Langevin dynamics for T control and Nosé-Hoover Langevin piston method for P control were used. NAMD output was stored every 20,000 steps rendering trajectories composed of 2500 frames which were processed and analyzed with VMD 1.9.2 [61] and Carma [62,63].

In order to evaluate the significance of structural change along MD simulations, initial and final geometries in every MD trajectory were structurally compared using three methods that follow completely different approaches to align structurally two proteins: (a) the rigid version of the *F*lexible structural *A*lignmen*T* by *C*haining *A*ligned fragment pairs allowing *T*wists (rigid *FATCAT*) algorithm [40], (b) the *C*ombinatorial *E*xtension (*CE*) algorithm [41], and the *TM* score-based *Align* (*TM-Align*) algorithm [42]. FATCAT and CE comparisons were obtained with the Protein Structure Comparison Tool v 4.2.0 downloaded from the PDB for use with local custom structure alignment [64] whereas TM-Align comparisons were obtained with the server https://zhanglab.ccmb.med.umich.edu/TM-align. According to the prescriptions given in the references for these methods, statistical significance in the structural relationship is considered as follows: (a) FATCAT *p*-value < 0.001, with lower values indicating higher similarity, (b) CE Z-score > 4.0, with larger values indicating stronger structural resemblance and Z-scores between 3.0 and 4.0 suggesting some structural similarity, and (c) TM-score closer to 1.0 for better similarities in the scale (0.0–1.0), with values between 0.40 and 0.50 suggesting some structural relationship.

## Figures and Tables

**Table 1:** Experimental structures of human saposins available in the PDB at 15 January 2018.

Type	PDB Id	Title	Struct.	Conf. ^1^	State	HET ^2^	Occ ^3^	Year	Ref.
Saposin A	2DOB	Human SapA	Xray	Closed	Monom	Ca^2+^	No	2006	[25]
	4UEX	Human SapA at lysosomal pH	Xray	Closed	Monom	-	Yes	2015	[26]
	4DDJ	SapA in complex with LDAO	Xray	Open	Dimer	LDAO	No	2012	[11]
Saposin B	1N69	Human SapB with PEH	Xray	Open	Dimer	PEH	No	2003	[13]
	4V2O	Human SapB in complex with CLQ	Xray	Open	Dimer	CLQ	Yes	2016	[27]
Saposin C	1M12	Human SapC	NMR	Closed	Dimer	-	No	2003	[28]
	1SN6	Human SapC in SDS micelles	NMR	Open	Monom	-	No	2005	[29]
	2GTG	Human SapC	Xray	Closed	Monom	-	No	2006	[25]
	2QYP	Orthorhombic crystal of SapC dimer	Xray	Open	Dimer	-	No	2008	[15]
	2Z9A	Human SapC dimer	Xray	Open	Dimer	-	No	2008	[15]
Saposin D	2RB3	Human SapD	Xray	Closed	Dimer	-	Yes	2008	[15]
	2R0R	K9E variant of human SapD	Xray	Closed	Dimer	SO_4_^2−^	Yes	2008	[15]
	2R1Q	Iodinated SapD in space group C222_1_	Xray	Closed	Dimer	IYR	No	2008	[15]
	3BQP	Orthorhombic human SapD	Xray	Closed	Monom	Mg^2+^	Yes	2008	[17]
	3BQQ	Triclinic human SapD	Xray	Closed	Monom	-	Yes	2008	[17]

^1^ Conformation, ^2^ Heterogroups present in the structure: LDAO = lauryldimethyl-N-oxide, PEH = di-stearoyl-3-SN-phosphatidyl ethanolamine, CLQ = chloroquine, IYR = 3-iodotyrosine, ^3^ Atoms with fractional occupancy in the electron density.

**Table 2:** Percent identity matrix for multiple sequence alignment of human saposins ^1^.

Type	SapA	SapB	SapC	SapD
SapA	100.0	23.08	39.74	36.71
SapB	23.08	100.0	16.00	22.37
SapC	39.74	16.00	100.0	34.18
SapD	36.71	22.37	34.18	100.0

^1^ Computed with Clustal Omega [32,33].

**Table 3:** Acidic and basic amino acids and total charge q_T_ of human saposins at different pH values.

Type	PDB Id	Conf.^2^	State	Number of					q_T_^3^	q_T_^1^ at		
				E	D	R	K	H		pH = 7.0	pH = 5.0	pH = 4.5
SapA	2DOB	Closed	Monom	8	7	1	6	0	−8	−8	−8	−3
	4UEX	Closed	Monom	8	7	1	6	0	−8	−8	−8	−2
	4DDJ	Open	Dimer1 ^4^	8	7	1	6	0	−8	−8	−6	−1
SapB	1N69	Open	Dimer2 ^4^	5	6	2	3	2	−6	−9 (−6, −5)	−6 (−4, −4)	+1 (−1, 0)
	4V2O	Open	Dimer2	5	6	2	3	2	−6	−10 (−6, −4)	−8 (−4, −4)	−2 (−1, −1)
	Model	Closed	Monom	5	6	2	3	2	−6	−6	−4	−1
SapC	1M12	Closed	Monom	11	5	1	7	1	−8	−8	−4	+2
	1SN6	Open	Monom	11	5	1	7	1	−8	−8	−6	+3
	2GTG	Closed	Monom	11	5	0	7	1	−9	−9	−8	−1
	2QYP	Open	Dimer2	11	5	0	7	1	−9	−17 (−9, −8)	−6 (−6, −6)	+5 (+4, +2)
	2Z9A	Open	Dimer2	11	5	0	7	1	−9	−18 (−9, −9)	−8 (−7, −6)	+1 (0, 0)
SapD	2RB3	Closed	Dimer2	8	4	1	7	0	−4	−8 (−4, −4)	−6 (−3, −3)	0 (0, −2)
	2R0R ^5^	Closed	Dimer2	9	4	1	6	0	−6	−12 (−6, −6)	−8 (−5, −5)	−2 (−2, −2)
	2R1Q	Closed	Dimer2	8	4	1	7	0	−4	−8 (−4, −4)	−2 (−3, −3)	+3 (0, 0)
	3BQP	Closed	Monom	8	5	1	7	0	−5	−5	−4	0
	3BQQ	Closed	Monom	8	5	1	7	0	−5	−5	−3	−1
	Model	Open	Monom	8	4	1	7	0	−4	−4	−2	+3

^1^ Computed with propka 3.1 [38,39]. For dimers, the first value is that computed for the whole dimer and values in parentheses are those computed separately for the two chains. ^2^ Conformation type. ^3^ Total charge at pH 7 resulting from the balance between acidic and basic residues (assuming uncharged histidines). ^4^ Dimer1 and dimer2 mean dimeric states with 1 and 2 chains, respectively, in the crystal structure. ^5^ Variant K9E.

**Table 4:** Significance scores of structural superpositions of initial and final structures in 100-ns MD simulations of open forms of human saposins computed three structural alignment methods.

pH	FATCAT Rigid		CE		TM-Align	
	*p*-Value	RMSD^1^	Z-Score	RMSD ^1^	TM-Score	RMSD ^1^
Saposin A						
7	4.02 × 10^−5^	6.48	4.25	5.75	0.407	3.41
5	2.08 × 10^−9^	3.71	4.74	3.99	0.579	3.26
4.5	1.01 × 10^−8^	4.00	4.58	4.22	0.563	2.62
Saposin B						
7	6.93 × 10^−12^	2.89	5.04	2.79	0.683	2.77
5	5.34 × 10^−11^	3.13	4.89	3.01	0.643	2.82
4.5	2.38 × 10^−9^	3.77	4.58	3.51	0.544	3.08
Saposin C						
7	3.22 × 10^−5^	5.87	3.89	2.81	0.463	3.39
5	1.40 × 10^−8^	3.09	4.89	2.66	0.686	2.83
4.5	2.44 × 10^−8^	3.21	4.89	3.44	0.582	3.29

^1^ Values in Å.

## Supplementary Materials

The following are available online at www.mdpi.com/xxx/link, Table S1: Surface pockets detected by DogSite in closed conformations of saposins, Table S2: Residues with fractional values of occupancy in the electron density in crystal structures of saposins. Figure S1: Structural alignment of homology-modeled structures of closed saposin B and open saposin D with their corresponding templates. Figure S2: Geometry of proline 39 in saposin A. Figure S3: Residues with fractional values of occupancy in the electron density of the crystal structure of saposin A.

## References

[1] Barral D.C., Brenner M.B. (2007). CD1 antigen presentacion: How it works. Nat. Rev. Immunol..

[2] Cohen N.R., Garg S., Brenner M.B. (2009). Antigen presentation by CD1 lipids, T cells, and NKT cells in microbial immunity. Adv. Immunol..

[3] Van Rhijn I., Godfrey D.I., Rossjohn J., Moody D.B. (2015). Lipid and small-molecule display by CD1 and MR1. Nat. Rev. Immunol..

[4] Kang S.J., Cresswell P. (2004). Saposins facilitate CD1-restricted presentation of an exogenous lipid antigen to T cells. Nat. Immunol..

[5] Zhou D., Cantu C., Sagiv Y., Schrantz N., Kulkarni A.B., Qi X., Mahuran D.J., Morales C.R., Grabowski G.A., Benlagha K. (2004). Editing of CD1d-bound lipid antigens by endosomal lipid transfer proteins. Science.

[6] Kolter T., Sandhoff K. (2005). Principles of lysosomal membrane digestion: Stimulation of sphingolipid degradation by sphingolipid activator proteins and anionic lysosomal lipids. Annu. Rev. Cell Dev. Biol..

[7] Meyer R.C., Giddens M.M., Coleman B.M., Hall R.A. (2014). The protective role of prosaposin and its receptors in the nervous system. Brain Res..

[8] Leon L., Tatituri R.V.V., Grenha R., Sun Y., Barral D.C., Minnaard A.J., Bhowruth V., Veerapen N., Besra G.S., Kasmar A. (2012). Saposins utilize two strategies for lipid transfer and CD1 antigen presentation. Proc. Natl. Acad. Sci. USA.

[9] Alattia J.R., Shaw J.E., Yip C.M., Privé G.G. (2006). Direct visualization of saposin remodeling of lipid bilayers. J. Mol. Biol..

[10] Schulze H., Sandhoff K. (2014). Sphingolipids and lysosomal pathologies. Biochim. Biophys. Acta.

[11] Popovic K., Holyoake J., Pomès R., Privé G.G. (2012). Structure of saposin a lipoprotein discs. Proc. Natl. Acad. Sci. USA.

[12] Yuan W., Qi X., Tsang P., Kang S.J., Ilarionov P.A., Besra G.S., Gumperz J., Cresswell P. (2007). Saposin B is the dominant saposin that facilitates lipid binding to human CD1d molecules. Proc. Natl. Acad. Sci. USA.

[13] Ahn V.E., Faull K.F., Whitelegge J.P., Fluharty A.L., Privé G.G. (2003). Crystal structure of saposin B reveals a dimeric shell for lipid binding. Proc. Natl. Acad. Sci. USA.

[14] Alattia J.R., Shaw J.E., Yip C.M., Privé G.G. (2007). Molecular imaging of membrane interfaces reveals mode of β-glucosidase activation by saposin C. Proc. Natl. Acad. Sci. USA.

[15] Rossmann M., Schultz-Heienbrok R., Behlke J., Remmel N., Allings C., Sandhoff K., Saenger W., Maier T. (2008). Crystal structures of human saposins C and D: Implications for lipid recognition and membrane interactions. Structure.

[16] Atrian S., López-Viñas E., Gómez-Puertas P., Chabás A., Vilageliu L., Grinberg D. (2008). An evolutionary and structure-based docking model for glucocerebrosidase-saposin C and glucocerebrosidase-substrate interactions: Relevance for Gaucher disease. Proteins.

[17] Popovic K., Privé G.G. (2008). Structures of the human ceramide activator protein saposin D. Acta Crystallogr. D.

[18] Winau F., Schwierzeck V., Hurwitz R., Remmel N., Sieling P.A., Modlin R.L., Porcelli S.A., Brinkmann V., Sugita M., Sandhoff K. (2004). Saposin C is required for lipid presentation by human CD1b. Nat. Immunol..

[19] Vartabedian V.F., Savage P.B., Teyton L. (2016). The processing and presentation of lipids and glycolipids to the immune system. Immunol. Rev..

[20] Malinina L., Patel D.J., Brown R.E. (2017). How α-helical motifs form functionally diverse lipid-binding compatments. Annu. Rev. Biochem..

[21] José-Estanyol M., Gomis-Rüth F.X., Puidomènech P. (2004). The eight-cysteine motif, a versatile structure in plant proteins. Plant Physiol. Biochem..

[22] Salcedo G., Sánchez-Monge R., Barber D., Díaz-Perales A. (2007). Plant non-specific lipid transfer proteins: An interface between plant defence and human allergy. Biochim. Biophys. Acta.

[23] Ng T.B., Cheung R.C.F., Wong J.H., Ye X. (2012). Lipid transfer proteins. Biopolymers.

[24] Tordesillas L., Cubells-Baeza N., Gómez-Casado C., Berin C., Esteban V., Barcik W., O’Mahony L., Ramirez C., Pacios L.F., Garrido-Arandia M. (2017). Mechanisms underlying induction of allergic sensitization by Pru p 3. Clin. Exp. Allergy.

[25] Ahn V.E., Leyko P., Alattia J.R., Chen L., Privé G.G. (2006). Crystal structures of saposins A and C. Protein Sci..

[26] Hill C.H., Read R.J., Deane J.E. (2015). Structure of human saposin A at lysosomal pH. Acta Crystallogr. F.

[27] Huta B.P., Mehlenbacher M.R., Nie Y., Lai X., Zubieta C., Bou-Abdallah F., Doyle R.P. (2016). The lysosomal protein saposin B binds chloroquine. Chem. Med. Chem..

[28] De Alba E., Weiler S., Tjandra N. (2003). Solution structure of human saposin C: PH-dependent interaction with phospholipid vesicles. Biochemistry.

[29] Hawkins C.A., De Alba E., Tjandra N. (2005). Solution structure of human saposin C in a detergent environment. J. Mol. Biol..

[30] Holm L., Laakso L.M. (2016). Dali server update. Nucl. Acids Res..

[31] Holm L., Sander C. (1995). Dali: A network tool for structure comparison. Trends Biochem. Sci..

[32] Sievers F., Wilm A., Dineen D., Gibson T.J., Karplus K., Li W., Lopez R., McWilliam H., Remmert M., Söding J. (2011). Fast, scalable generation of high-quality protein multiple sequence alignments using Clustal Omega. Mol. Syst. Biol..

[33] Li W., Cowley A., Uludag M., Gur T., McWilliam H., Squizzato S., Park Y.M., Buso N., Lopez R. (2015). The EMBL-EBI bioinformatics web and programmatic tools framework. Nucl. Acids Res..

[34] Volkamer A., Griewel A., Grombacher T., Rarey M. (2010). Analyzing the topology of active sites: On the predictions of pockets and sub-pockets. J. Chem. Inf. Model..

[35] Volkamer A., Kuhn D., Grombacher T., Rippmann T., Rarey M. (2012). Combining global and local measures for structure-based druggability predictions. J. Chem. Inf. Model..

[36] Johanssom M.U., Zoete V., Michielin O., Guex N. (2012). Defining and searching for structural motifs using DeepView/Swiss-PdbViewer. BMC Bioinform..

[37] Qi X., Grabowski G.A. (2001). Differential membrane interactions of saposins A and C: Implications for the functional specificity. J. Biol. Chem..

[38] Olsson M.H.M., Sondergaard C.R., Rostkowski M., Jensen J.H. (2011). Propka 3: Consistent treatment of internal and surface residues in empirical pKa predictions. J. Chem. Theory Comput..

[39] Sondergaard C.R., Olsson M.H.M., Rostkowski M., Jensen J.H. (2011). Improved treatment of ligands and coupling effects in empirical calculation and rationalization of pKa values. J. Chem. Theory Comput..

[40] Ye Y., Godzik A. (2003). Flexible structure alignment by chaining aligned fragment pairs allowing twists. Bioinformatics.

[41] Shyndyalov I.N., Bourne P.E. (1998). Protein structure alignment by incremental combinatorial extension (CE) of the optimal path. Protein Eng..

[42] Zhang Y., Skolnick J. (2005). TM-Align: A protein structure alignment algorithm based on the TM-score. Nucl. Acids Res..

[43] Vaccaro A.M., Ciaffoni F., Tatti M., Salvioli R., Barca A., Tognozzi D., Scerch C. (1995). pH-dependent conformational properties of saposins and their interactions with phospholipid membranes. J. Biol. Chem..

[44] John M., Wendeler M., Heller M., Sandhoff K., Kessler H. (2006). Characterization of human saposins by NMR spectroscopy. Biochemistry.

[45] Ciaffoni F., Tatti M., Boe A., Salvioli R., Fluharty A., Sonnino S., Vaccaro A.M. (2006). Saposin B binds and transfers phospholipids. J. Lipid Res..

[46] Klein A., Henseler M., Klein C., Suzuki K., Harzer K., Sandhoff K. (1994). Sphingolipid activator protein (sap-D) stimulates the lysosomal degradation of ceramide in vivo. Biochem. Biophys. Res. Commun..

[47] Linke T., Wilkening G., Sadeghlar F., Mozcall H., Bernardo K., Schuchman E., Sandhoff K. (2001). Interfacial regulation of acid ceramidase activity. Stimulation of ceramide degradation by lysosomal lipids and sphingolipid activator proteins. J. Biol. Chem..

[48] Ciaffoni F., Salvioli R., Tatti M., Arancia G., Crateri P., Vaccaro A.M. (2001). Saposin D solubilizes anionic phospholipid-containing membranes. J. Biol. Chem..

[49] (2017). The PyMOL Molecular Graphics System.

[50] Arnold K., Bordoli L., Kopp J., Schwede T. (2006). The SWISS-MODEL Wokspace: A web-based environment for protein structure modelling. Bioinformatics.

[51] Biasini M., Bienert S., Waterhouse A., Arnold K., Studer G., Schmidt T., Kiefer F., Gallo Cassarino T., Bertoni M., Bordoli L. (2014). SWISS-MODEL: Modelling protein tertiary and quaternary structure using evolutionary information. Nucl. Acids Res..

[52] Dolinsky T.J., Nielsen J.E., McCammon J.A., Baker N. (2004). PDB2PQR: An automated pipeline for the setup, execution, and analysis of Poisson-Boltzmann electrostatic calculations. Nucl. Acids Res..

[53] Dolinsky T.J., Czodroowski P., Li H., Nielsen J.E., Jensen J.H., Klebe G., Baker N.A. (2007). PDB2PQR: Expanding and upgrading automated preparation of biomolecular structures for molecular simulations. Nucl. Acids Res..

[54] McKerell A.D., Bashford D., Bellott M., Dunbrack R.L., Evanseck J., Field M.J., Fischer S., Gao J., Guo H., Ha S. (1998). All-atom empirical potential for molecular modeling and dynamics studies of proteins. J. Phys. Chem. B.

[55] Baker N., Sept D., Joseph S., Holst M.J., McCammon J.A. (2001). Electrostatics of nanosystems: Applications to microtubules and the ribosome. Proc. Natl. Acad. Sci. USA.

[56] Jurrus E., Engel D., Star K., Monson K., Brandi J., Felberg L.E., Brookes D.H., Wilson L., Chen J., Liles K. (2017). Improvements to the APBS biomolecular solvation software suite. Protein Sci..

[57] Best R.B., Zhu X., Shim J., Lopes P.E.M., Mittal J., Feig M., MacKerell A.D. (2012). Optimization of the additive CHARMM all-atom protein force field targeting improved sampling of the backbone phi, psi and side-chain chi1 and chi2 dihedral angles. J. Chem. Theory Comput..

[58] Phillips J.C., Braun R., Wang W., Gumbart J., Tajkhorshid E., Villa E., Chipot C., Skeel R.D., Kalé L., Schulten K. (2005). Scalable molecular dynamics with NAMD. J. Comput. Chem..

[59] Jorgensen W.L., Chandrasekhar J., Madura J.D., Impey R.W., Klein M.L. (1983). Comparison of simple potential functions for simulating liquid water. J. Chem. Phys..

[60] Darden T., York D., Pedersen L. (1993). Particle mesh Ewald: An N log(N) method for Ewald sums in large systems. J. Chem. Phys..

[61] Humphrey W., Dalke A., Schulten K. (1996). VMD—Visual molecular dynamics. J. Mol. Graph..

[62] Glykos N.M. (2006). Carma: A molecular dynamics analysis program. J. Comput. Chem..

[63] Koukos P.I., Glykos N.M. (2013). Grcarma: A fully automated task-oriented interface for the analysis of molecular dynamics trajectories. J. Comput. Chem..

[64] Prlic A., Bliven S., Rose P.W., Bluhm W.F., Bizon C., Godzik A., Bourne P.E. (2010). Pre-calculated protein structure alignments at the RCSB PDB website. Bioinformatics.

