# Supplementary material for: A Comparative Study of Human Saposins
Source: Molecules. 2018 Feb 14;23(2):422. doi: 10.3390/molecules23020422 (PMC6017893; doi:10.3390/molecules23020422)
Supplement: Supplementary file 1 [file molecules-23-00422-s001.pdf]

Comparative study of human saposins

María Garrido-Arandia, Bruno Cuevas-Zuviría, Araceli Díaz-Perales, and Luis F. Pacios*

Corresponding author:
e-mail: luis.fpacios@upm.es

Supplementary Material

Contents	Page
Table S1	S2
Table S2	S3
Figs. S1 & S2	S4
Fig. S3	S5

Table S1. Surface pockets detected by DogSite in the closed conformations of human saposins. Pocket numbering corresponds to their arrangement in order of decreasing volume.

	Saposin A (4UEX)			Saposin B (model)	Saposin C (2GTG)				Saposin D (2RB3)			
Pocket:	P0	P1	P2	P0	P0	P1	P2	P3	P0	P1	P2	P3
V (Å^3)	195.8	178.5	130.8	804.4	202.1	156.5	142.4	130.8	137.9	135.1	127.5	120.0
S (Å^2)	381.5	477.3	362.2	1521	485.1	259.7	427.1	323.1	409.0	319.1	284.1	393.3
Drug score [1]	0.48	0.41	0.26	0.85	0.30	0.35	0.34	0.26	0.30	0.18	0.27	0.27
Residues [2]	L2	G15	E24	V3 C4 D6	E9	L11	E14	T16	V57	L32	Y14	D16
	D5	L18	E25	C7 I8 M10	V12	V12	K17	I19	E60	E33	L15	K21
	I6	K19	L28	V11 T12 Q15	K13	V15	L18	D20	I61	K34	N18	N22
	D9	N21	V51	F24 A27 L28	P68	L29	N21	L62	E64	G35	L19	S23
	V10	A22	D52	H31 V32 K33	E69	F32	K34	E65	F70	C36	A30	T24
	Y30	T23	L55	E35 C36 D37	L70	D33	M35	V66	K74	S37	A31	K25
	L31	E24	P56	M43 K48 Y50	V71	Q48	K38	S67	I75	Q44	K34	L62
	T34	I62		I51 S52 Y54	C72	V50		P68		D48	G35	V63
	C35	K63		S55 E56 I57	S73	V51				V51	F38	E64
	L38	E65		A58 I59 M61	C78	D52				A52		V65
	M43	M66		M62 H64 M65	S79	Y54				E55		M66
	C47	S67		I70 V74 F76		G55						
						S56						
						I58						

[1] Druggability score in DogSite is based on a linear combination of pocket descriptors such as volume, hydrophobicity, and enclosure. See Ref. 35 in main text for details. [2] Residues in the spatial domain of each surface pocket

Table S2. Residues with fractional values of occupancy in the electron density in crystal structures of human saposins [1]

Saposin A			Saposin B			Saposin D											
4UEX [2]			4V2O [3]			2RB3 [4]			2R0R			3BQP [2]			3BQQ [5]		
E25 A	0.55	0.45	Q5 A	0.50	0.50	K74 A	0.50	0.50	E64 A	0.50	0.50	L11 A	0.50	0.50	E53 A	0.50	0.50
M66 A	0.54	0.46	R38 B	0.50	0.50	V57 B	0.50	0.50				Y14A	0.50	0.50	K74 A	0.50	0.50
E71 A	0.49	0.51	Q5 C	0.50	0.50	E60 B	0.50	0.50				L15 A	0.50	0.50	E6 B	0.50	0.50
K19 B	0.49	0.51				E64 B	0.50	0.50				I28 A	0.50	0.50	Q44 B	0.50	0.50
E24 B	0.47	0.53				S69 C	0.50	0.50				Q44 A	0.50	0.50	E53 B	0.50	0.50
E25 B	0.47	0.53				K74 C	0.50	0.50				M66 A	0.50	0.50	R17 C	0.50	0.50
K33 B	0.57	0.43				E53 D	0.50	0.50				L11 B	0.50	0.50	E53 C	0.50	0.50
W37 B	0.50	0.50										L15 B	0.50	0.50	K74 C	0.50	0.50
N42 B	0.45	0.55										L19 B	0.50	0.50	E60 D	0.50	0.50
S46 B	0.50	0.50										M66 B	0.50	0.50	K74 D	0.50	0.50
I50 B	0.34	0.66										S69 B	0.50	0.50			

[1] Residue, chain, and two occupancy factors in the order given in pdb structure files [2] Monomer. Two chains (A,B) in the asymmetric unit [3] Dimer. Three chains (A,B,C) in the asymmetric unit [4] Dimer. Four chains (A,B,C,D) in the asymmetric unit [5] Monomer. Four chains (A,B,C,D) in the asymmetric unit

Supplementary figures

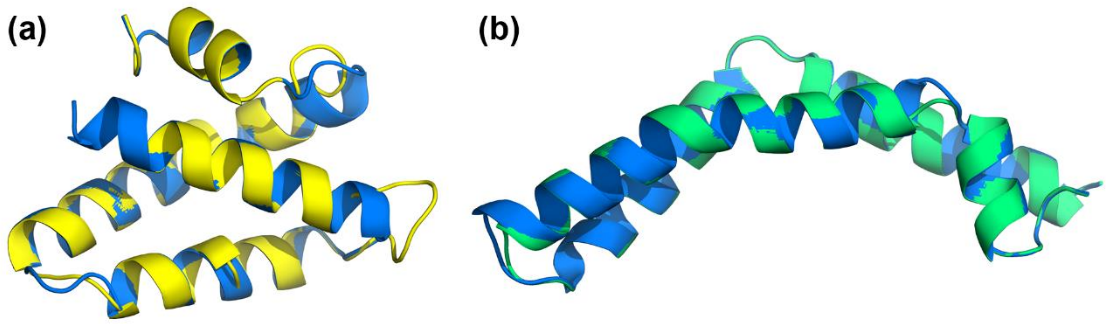

Figure S1. Structural alignment of homology-modeled structures of closed saposin B and open saposin D with their corresponding templates. **(a)** Closed form of saposin B (yellow) modeled using the crystal closed structure of saposin A (blue: chain A in 4UEX) as template. RMSD backbone = 0.716 Å with 72/78 residues in the superposition **(b)** Open form of saposin D (green) modeled using the crystal open structure of saposin A (blue: 4DDJ) as template. RMSD backbone= 0.293 Å with 77/78 residues in the superposition.

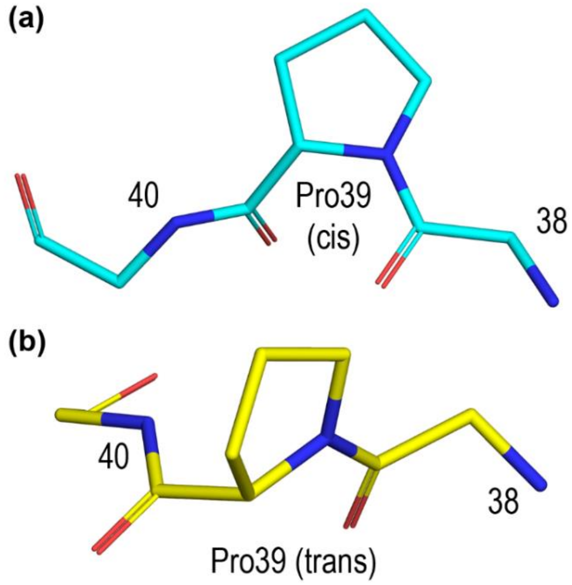

Figure S2. Geometry of proline 39 in the backbone of saposin A. **(a)** Closed conformation (chain A in 4UEX). **(b)** Open conformation (4DDJ).

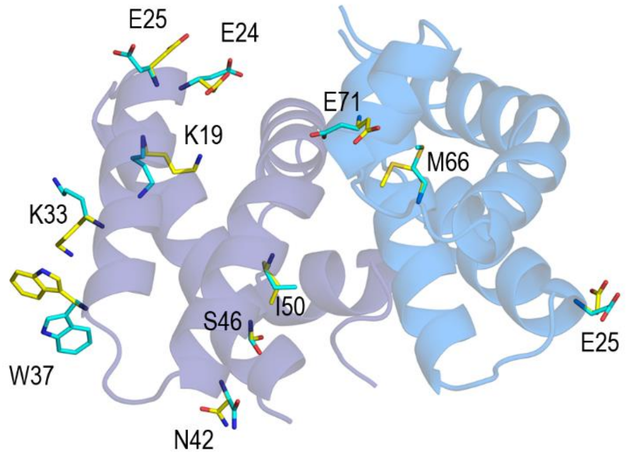

Figure S3. Residues having atoms with fractional values of occupancy in the electron density for the closed form of human saposin A. The two chains in the asymmetric unit in the crystal structure 4UEX are colored in marine blue (chain A) and deep blue (chain B). Carbons in the two side chain conformations that correspond to the two occupancy fractional values are colored in yellow (3 residues in chain A) and cyan (8 residues in chain B). See Table S2.
